# The Reconstruction of the Metropolitan Asylums Board

**Published:** 1915-01-16

**Authors:** 


### THE RECONSTRUCTION OF THE METROPOLITAN ASYLUMS BOARD.

The Report of the Law and Parliamentary Committee of the Metropolitan Asylums Board upon the constitution of the Board, presented to and adopted at a recent meeting of that body, shows that the importance of the centralisation and co-ordination of the different public services concerned with the treatment and prevention of sickness is making headway, slow though the progress is, amongst the authorities dealing with the work. The Asylums Board consists of fifty-five managers elected by the various Metropolitan Boards of Guardians from their members, and eighteen nominated by the Local Government Board. In their report the committee say they are not prepared at present to submit any formal recommendation to the Board. Nevertheless they are of opinion that in view of the close relationship between certain of the duties of the managers and of the City and Borough Councils, it is desirable that the constitution of the Board should be revised so as to permit of the representation of the Councils thereon.

This is a step in the right direction, but it deals with only a minute portion of a much larger problem. The public medical work of the Metropolis is at present controlled by twenty-nine City and Borough Councils, twenty-eight Boards of Guardians, the Metropolitan Asylums Board, and the London County Council. By far the greater part of this work is being dealt with from a purely local standpoint, without any regard to the needs or achievements of adjacent areas. Even that part which is centralised under the Metropolitan Asylums Board and the London County Council is being carried on without any close co-operation with the local authorities. It is not, therefore, surprising to find that there is much overlapping, much difference in the efficiency with which the work is carried out in the various localities, and the lack of many facilities which could be easily supplied under a more rational system of administration. We find the Metropolitan Asylums Board and the London County Council asserting that the local authorities are not making full use or the best use of the special institutions provided, and we find the local authorities retorting that these central institutions have been provided and specialised without a full consideration of local requirements.

There is undoubtedly truth in both contentions. Sir Thomas Duncombe Mann drew attention to the matter in his evidence before the Royal Commission on the Poor Laws and Relief of Distress. He said, "Some duplication of work and of expenditure is unfortunately unavoidable with the present overlapping of duties and division of authority between various Metropolitan bodies. This is also a source of friction between the authorities when one body, being naturally without experience and inside knowledge of the working and administration of another, becomes querulous with regard to the performance of that other body's duties." Mr. James T. Helby, then Chairman of the Board, giving evidence before the same Commission, expressed similar views and indicated the solution. He suggested the abolition of the Metropolitan Asylums Board and the Boards of Guardians, and the formation of a central Poor-Law body for London, consisting partly of elected members and partly of members nominated by the Local Government Board and the London County Council. This central body was to control all the Poor-Law institutions in its area, whilst outdoor relief was to be in the hands of small local committees appointed by the central body. It will be remembered that both the majority and minority reports of the Commission also recommended the abolition of Boards of Guardians and the substitution of one authority to deal with the whole of the Poor-Law work of the Metropolis. This was over five years ago, and we are still waiting for these reforms to be carried out. In the intervening years many improvements have been made, and many are still possible without radical changes in administration; but the medical service of the Poor-Law infirmaries will never reach the high standard of efficiency of the voluntary hospitals until the formation of one central body allows the problem to be dealt with as a whole.

